# Whole Three-Dimensional Dosimetry of Carbon Ion Beams with an MRI-Based Nanocomposite Fricke Gel Dosimeter Using Rapid *T*_1_ Mapping Method

**DOI:** 10.3390/gels7040233

**Published:** 2021-11-25

**Authors:** Shinya Mizukami, Yusuke Watanabe, Takahiro Mizoguchi, Tsutomu Gomi, Hidetake Hara, Hideyuki Takei, Nobuhisa Fukunishi, Kenichi L. Ishikawa, Shigekazu Fukuda, Takuya Maeyama

**Affiliations:** 1 School of Allied Health Sciences, Kitasato University, Sagamihara 252-0373, Japan; shinmiz@kitasato-u.ac.jp (S.M.); y-nabe@kitasato-u.ac.jp (Y.W.); gomi@kitasato-u.ac.jp (T.G.); harah@kitasato-u.ac.jp (H.H.); 2 Graduate School of Medical Sciences, Kitasato University, Sagamihara 252-0373, Japan; ar17859@st.kitasato-u.ac.jp; 3 Proton Medical Research Center, University of Tsukuba Hospital, Tsukuba 305-8576, Japan; hide.y.takei@gmail.com; 4 Nishina Center for Accelerator-Based Science, RIKEN, Saitama 351-0198, Japan; fukunisi@ribf.riken.jp; 5 Department of Nuclear Engineering and Management, Graduate School of Engineering, The University of Tokyo, Tokyo 113-8656, Japan; ishiken@n.t.u-tokyo.ac.jp; 6 QST Hospital, National Institutes for Quantum Science and Technology, Chiba 263-8555, Japan; fukuda.shigekazu@qst.go.jp; 7 Department of Chemistry, School of Science, Kitasato University, Sagamihara 252-0373, Japan

**Keywords:** heavy ion beam dosimetry, gel dosimeter, nanocomposite Fricke, linear energy transfer, MRI, variable flip angle

## Abstract

MRI-based gel dosimeters are attractive systems for the evaluation of complex dose distributions in radiotherapy. In particular, the nanocomposite Fricke gel dosimeter is one among a few dosimeters capable of accurately evaluating the dose distribution of heavy ion beams. In contrast, reduction of the scanning time is a challenging issue for the acquisition of three-dimensional volume data. In this study, we investigated a three-dimensional dose distribution measurement method for heavy ion beams using variable flip angle (VFA), which is expected to significantly reduce the MRI scanning time. Our findings clarified that the whole three-dimensional dose distribution could be evaluated within the conventional imaging time (20 min) and quality of one cross-section.

## 1. Introduction

In radiotherapy, it is very important to improve the accuracy of the radiation dose based on the three-dimensional (3D) shape and position of the tumor target, thus maximizing the dose to the target while minimizing damage to the adjacent normal tissue. To fulfill this need using linear accelerators in external radiotherapy, irradiation techniques, such as intensity-modulated radiotherapy (IMRT), stereotactic radiosurgery (SRS), and stereotactic body radiotherapy (SBRT), are performed in many clinical facilities. Heavy ion and proton beams with the Bragg peak are expected to reduce adverse events in normal tissues and improve treatment outcomes using beams with a more concentrated radiation dose [1]. In addition, carbon ions have a high linear energy transfer (LET) and various biological advantages over protons and photons [2,3,4].

While irradiation technology is advancing, dose administration in clinical settings needs to be of high precision and accuracy when performing complex processes such as tumor imaging and treatment planning. This is especially true when it comes to heavy ion and proton beams because a slight setup error can highly affect the dose distribution in the patients’ body, and thus, cause significant errors. In general, the dose distribution calculation before treatment and the setup of the treatment equipment throughout the treatment planning process are almost automated. However, this automation can induce errors that are very difficult to identify. For instance, the accuracy of dose distribution calculations and the ambiguity of the dose due to equipment setup errors can be major issues. Therefore, it is important to clarify potential dose distribution errors that may be caused by the selected dose calculation method and the equipment before treatment, and to verify the subsequent dose distribution to maintain irradiation accuracy [5]. Proton beams deliver almost no dose behind the distal end of the Bragg peak. In contrast, carbon beams have a high peak to plateau ratio, and an extra dose of approximately 10% is observed at the end of the range due to spallation. In addition, dose distribution measurements in carbon beams must be performed carefully because the lateral dose distribution is steep as a result of less multiple scattering.

Various types of detectors are used for carbon beam dosimetry [6]. Ionization chambers are recommended and widely adopted by the international atomic energy agency (IAEA) Technical Report Series No. 398 (TRS-398) [7,8,9]. Ionization chamber arrays and semiconductor arrays are also useful tools, but the spacing between detectors induces a distinct effect on the respective spatial resolution. Furthermore, radiochromic film has the advantage of high spatial resolution, but carbon ions have a much higher LET compared to protons, and thus, the use of film for carbon beam dosimetry is characterized by significant LET-dependent problems [10,11,12]. While 3D dose distribution measurements are necessary for accommodating the characteristics of the steep dose distribution of carbon beams, these tools are currently limited to point or two-dimensional (2D) measurements, and their use is limited for each quality assurance item.

In recent years, gel dosimeters, which are chemical dosimeters, have been attracting significant attention as promising tools for measuring 3D dose distributions [13,14,15,16,17,18,19,20,21]. Various forms of chemical dosimeters are used, such as the Fricke gel dosimeter [22,23,24], which uses the oxidation reaction of iron ions, and the polymer gel dosimeter [25,26,27], which uses radiation-induced polymerization in a monomer solution. The 3D spatial information of these methods is preserved by implanting radiosensitive chemicals in a spatially stable gel matrix. The chemical changes of the Fricke gel dosimeter and polymer gel dosimeter due to irradiation can be measured from their nuclear magnetic resonance (NMR) relaxation characteristics.

Several researchers have reported on the use of polymer gel dosimeters for carbon beam dosimetry [28,29,30,31]. However, polymer gel dosimeters are also LET-dependent, and thus, it is challenging to measure the dose distribution of carbon ion beams. While LET-dependent correction methods using dose-weighted sensitivity, calculated from Monte Carlo simulations, have been proposed [31], complex dose calibration is required. Maeyama et al. [32], developed a novel nanocomposite Fricke gel (NC-FG) with nano-sized clay particles and degassing operation to improve LET dependence. The carbon beam depth dose distribution was found to be in good agreement with the ionization chamber measurements [32,33,34].

The Fricke gel dosimeter can determine the dose distribution by dose-converting the *R*_1_ map, which is the reciprocal of the longitudinal relaxation time (*T*_1_), also known as the spin–lattice relaxation time. Magnetic resonance (MR)-based gel dosimeters affect the ultimate goal of dosimetry accuracy due to the uncertainty of MR scans. Since the optimized scan protocol provides improved measurement accuracy [35], several improvements to the MR imaging method for gel dosimeters have been reported. Vandecasteele and De Deene [36] quantified the relative contributions of error factors for dosimetry in polymer gel dosimeters, such as magnetic field strength, magnetic field inhomogeneity, and gel temperature during scanning. Papoutsaki et al. [37] used a multi-echo single-shot turbo spin echo pulse sequence for *T*_2_ measurements of polymer gel dosimeters to significantly reduce the imaging time. Cho et al. [38] succeeded in shortening the imaging time by using a high-speed spin echo MR imaging sequence in order to detect signal intensity changes in the Fricke gel dosimeter for gamma rays of a gamma knife.

The gel dosimeter itself acts as a phantom and provides 3D dose distribution data. The evaluation 2D cross-section acquired by MR measurements is imaged, compared, and verified with an ionization chamber and the treatment planning system. Therefore, only a part of the 3D dose data of the gel dosimeter is evaluated, and data other than the imaged cross-sections cannot be referred to. Furthermore, it is necessary to take an image for each evaluation cross-section, something which can be significantly time-consuming due to the plurality of cross-sections. Thus, whole 3D dose distribution using an MR scan has never been attempted [25], and this is a challenging issue for all MR-based gel dosimeters. In recent years, by using optical computed tomography (OCT) instead of MR imaging, whole three-dimensional dose distribution measurements have been performed [39,40,41,42]. However, the gel container shape and type of gel dosimeter are limited especially for ion beam irradiation. The advantages and disadvantages of MRI and OCT were summarized in the literature [43].

In this study, we investigated the whole three-dimensional dosimetry of carbon ion beam gel dosimetry by applying a new rapid MR imaging method. The rapid and accurate measurement of *T*_1_ relaxation time also remains an important goal in clinical examinations. Low-noise, high-resolution 3D mapping of *T*_1_ relaxation times could not be achieved in a clinically acceptable time frame (<30 min). Recently, the variable flip angle spoiled gradient recalled echo (VFA-SPGR) approach demonstrated a significant reduction in imaging time [44,45], similar to the ones achieved using conventional methods of inversion recovery [46,47,48,49]. By applying VFA-SPGR to ion beam gel dosimetry, the results show a successful reduction in the MRI scanning time. Furthermore, it was also demonstrated that the whole three-dimensional dose distribution could be roughly evaluated within the conventional imaging time (20 min) and the quality of one cross-section.

## 2. Materials and Methods

### 2.1. Gel Preparation

Nanocomposite Fricke gel (NC-FG) was prepared from 2% (*w*/*w*) nanoclay (synthetic hectorite or Laponite XLG; Na^+0.7^[(Si_8_Mg_5.5_Li_0.3_) O_20_(OH)_4_]^−0.7^, Rockwood Ltd., Widnes, UK, CAS No. 53320-86-8), 1 mM ammonium iron (II) sulfate hexahydrate (Fluka, Japan, CAS No. 7783-85-9), and 98% (*w*/*w*) ultra-pure water. NC-FG (375 g) was prepared as follows. First, 300 mL of deaerated ultra-pure water, obtained by bubbling N_2_ gas for 60 min, was mixed with nanoclay (7.5 g) with a magnetic stirrer for 60 min in an anaerobic glove box. The dispersion was then mixed with Fricke stock solution, consisting of 144.1 mg ammonium iron (II) sulfate hexahydrate and 75 mL deaerated ultra-pure water, for 60 min. The prepared NC-FG was sealed in five screw-cap borosilicate glass vials (LABORAN 50 mL screw-cap tube bottle, 9-852-09, AS-ONE, Osaka, Japan,) using copper (Cu) foil (Nitoms, copper foil tape J3160) under a pure argon atmosphere in a glove box. The NC-FG gelled over time due to its favorable thixotropic properties. In order to suppress the formation of bubbles and the formation of non-uniform *R*_1_ distribution during sealing, the vial was stirred together with the container with a rotation/revolution mixer (V-mini300, EME Corporation, Tokyo, Japan) for 3 min, and left to solidify for a day. The reference gel samples were prepared using 0–0.5 mM Fe^3+^ (Fe_2_(SO_4_)_3_·nH_2_O) (FUJIFILM Wako Pure Chemical Corporation, Japan, CAS No. 15244-10-7) and 2 wt% nanoclay. We evaluated the *R*_1_ per millimolar of iron from the slope of the calibration curve obtained from the measurements of the reference gel.

### 2.2. Irradiation

The irradiation experiments were performed at the Biological Irradiation Port of the Heavy Ion Medical Accelerator in Chiba (HIMAC) at the National Institutes for Quantum and Radiological Science and Technology, Japan on 5 July 2020. Two kinds of the ^12^C^6+^ beam at 290 MeV/u with a 10-cm diameter beam with an irradiation field of ±5% lateral dose uniformity and a pencil beam (σ = 27.6 mm) were used. The energies of both ion beams were attenuated with an energy absorber made of polymethyl methacrylate (PMMA) plates to adjust the range of the ion beams to the center of the 50 mL glass vials. The water equivalent thicknesses of the energy absorber for the 10-cm diameter beam and pencil beam irradiation were 99.83 and 104.39 mm H_2_O, respectively. In addition, the pencil beam was collimated using a brass collimator with a 10 × 10 mm^2^ square opening [50]. The 50 mL glass vials (φ35 × 78 mm) containing NC-FG were irradiated from the bottom at room temperature (25 °C). The bottom thickness of the glass vial was 1.3 ± 0.3 mm. The absorbed dose (referred to as the entrance surface dose, ESD hereafter) varied from 0 to 600 Gy. The ESD was calibrated by measuring the dose at the same position as the bottom surface of the sample using a Markus ionization chamber (IC) and a secondary electron monitor that was installed permanently on the upstream side of the beamline [51,52]. For comparison with gel dosimeters, the depth−dose profile of this carbon ion beam was also measured using the Markus IC by decreasing the beam energy using a binary filter-type range shifter consisting of plastic (PMMA) plates [50,53].

### 2.3. MRI Measurements

The longitudinal MR relaxation rate *R*_1_ (=1/*T*_1_) values were measured using the variable flip angle spoiled gradient recalled echo (VFA-SPGR) as a 3D imaging method. In this method, the *R*_1_ values were calculated from the difference in signal intensity using multiple flip angles, as shown below [44,45,54,55].

The signal intensity (*S_i_*) acquired at a flip angle (*α_i_*) is a function of the longitudinal relaxation time (*T*_1_), repetition time (*TR*), and equilibrium magnetization (*M*_0_), where *E*_1_ = exp(−*TR*/*T*_1_).

(1) $$S_{i}=\frac{M_{0}\left(1-E_{1}\right)\sin\left(\alpha_{i}\right)}{1-E_{1}\cos\left(\alpha_{i}\right)}$$

Signal acquisition at different flip angles allows for the determination of *T*_1_ by the following two equations.

(2) $$\frac{S_{i}}{\sin\left(\alpha_{i}\right)}=E_{1}\frac{S_{i}}{\tan(\alpha_{i})}+M_{0}\left(1-E_{1}\right)$$

(3) $$T_{1}=-\frac{TR}{ln\left(E_{1}\right)}$$

The usual inversion recovery (IR) method requires a long Repetition Time (*TR*), which increases the imaging time. In contrast, the VFA-SPGR method uses a short *TR*, and thus, the imaging time can be shortened [56]. In this study, a 3T MRI scanner (MAGNETOM Skyra, Siemens Medical Solutions, Erlangen, Germany) was used with a body array coil as the receiving coil. The 50-mL glass vials containing NC-FG were fixed in the center of the static magnetic field of the MRI, horizontal to the direction of the static magnetic field. The receiving coil was fixed using a spacer so that the NC-FG dosimeter could be replaced without changing the position of the receiving coil, and each irradiated gel sample was imaged at the same position. The imaging cross-section was sagittal and included the entire vial. Imaging was performed by the Turbo FLASH sequence at room temperature (21 °C). Table 1 provides a more detailed description of the imaging parameters used. The spin echo (SE) method was also used for a comparison with the VFA-SPGR method.

## 3. Results and Discussion

### 3.1. Whole R_1_ Distribution of NC-FG Using VFA Methods

**Figure 1:**
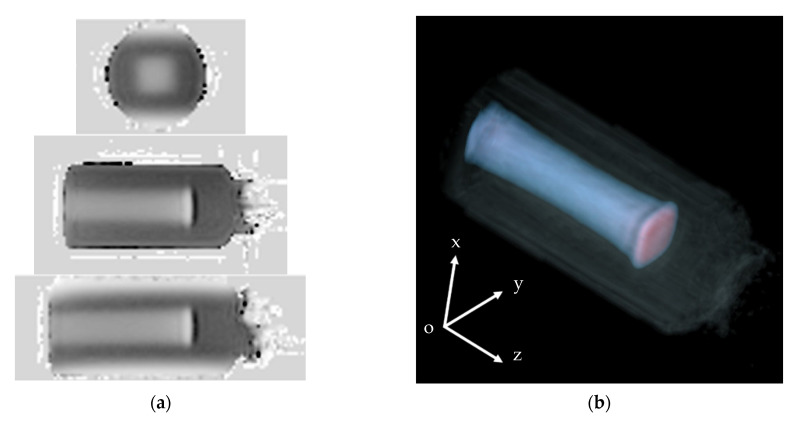
3D image of NC-FG-irradiated 450 Gy ESD obtained from MRI imaging. (**a**) Using MPR, (**b**) using volume rendering (VR). The upper, center, and lower panels in (**a**) are xy, yz, and xz views, respectively.

Figure 1 shows the longitudinal MR relaxation rate (*R*_1_ = 1/*T*_1_) 3D map of the NC-FG that was irradiated with a collimated 10 × 10 mm^2^ square pencil beam. This map was constructed from 40 images that were taken along the axes of the beams. Figure 1a demonstrates the *R*_1_ volume data (scanning plane, long axial plane, short axial plane) displayed by multi-planer reconstruction (MPR) (ADWIN version 6.4; GE). The imaging conditions were as follows: FOV = 256, spatial resolution = 1 mm, slice thickness = 1 mm, as shown in Table 1. In addition, the volume rendering (VR) images [57] are shown in Figure 1b for better viewing by the user (video and 3D data are included in Supplementary Materials, Video S1). The coloring display condition in the VR image is from blue to red, and the dose is increasing. As also shown in Supplementary Materials, Video S1, the image can be rotated and viewed from different angles. Our results clearly show that a square shape was maintained from the incident surface to the Bragg peak, and the beam traveled in a straight line and spread laterally near the peak. Specifically, the lateral beam extension shows the reduction from the entrance plane towards the Bragg peak, where it gets wider again. This phenomenon seems to be similar to that shown in the lateral dose distribution map of proton beams reported by Suit et al. [58] (shown in Figure 4 in reference [58]). The region of the flat dose near the entrance surface in the depth–dose profile, called the plateau [59], is not strictly flat. The depth–dose curve shows a declining trend once from the entrance surface dose, and then increases at the Bragg peak. This phenomenon is due to the decrease in the number of particles due to the spallation and the lateral beam spreading. The pencil beam used in this study was a 290 MeV/u carbon ion beam, adjusted by inserting an energy absorber made of 104.39 mm H_2_O PMMA, and collimated using a brass collimator with a 10 × 10 mm^2^ square opening. Thus, it was speculated that the beam spreading and generating secondary particles were more complicated. Further investigations using a simulation such as the PHTIS code [60,61,62] are required.

**Figure 2:**
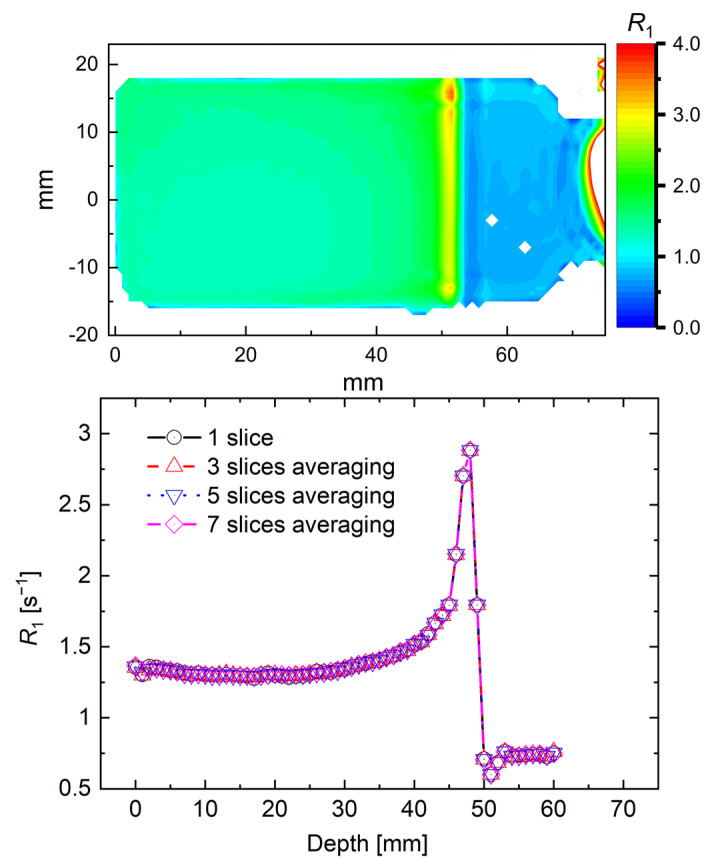
*R*_1_ distribution measured with VFA methods after irradiation with a 290 MeV/u carbon beam for NC-FG at 600 Gy ESD dose. Upper panel: 2D map, lower panel: line profile obtained from the 2D map.

**Figure 3:**
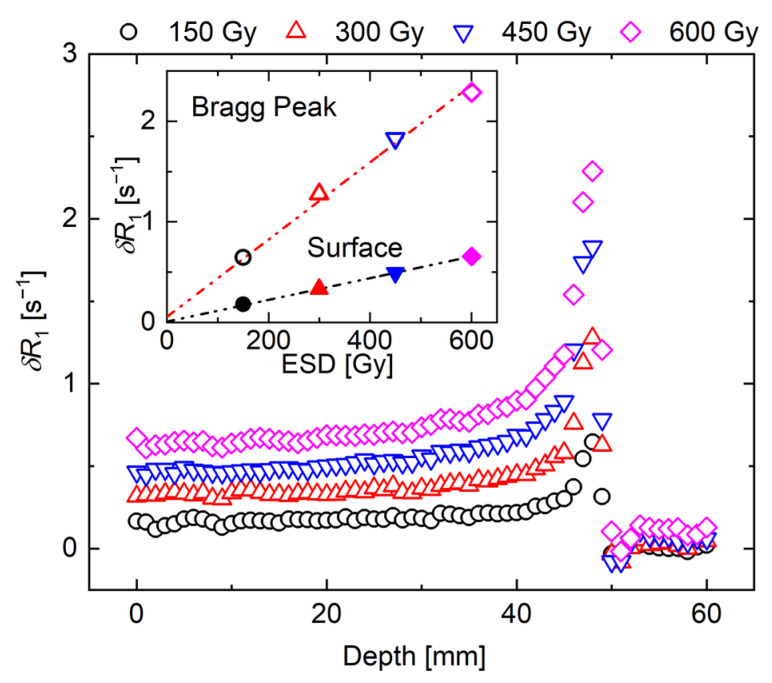
One-line depth-δ*R*_1_ distribution in NC-FG dosimeter. The inset shows *R*_1_ values [s^−1^] as a function of the ESD [Gy] at the entrance surface (solid symbol) and at the Bragg peak (open symbol). The ESD values are 150 (circle), 300 (upper triangle), 450 (lower triangle), and 600 (rhombus) Gy.

One cross-section and its distribution obtained from a single pixel (1 mm) line is shown in Figure 2, in the upper and lower panels, respectively. The distribution obtained by averaging the adjacent three, five, and seven slices is also shown in the lower panel. The standard deviation (SD) of the *R*_1_ value within seven slices is 1.30 ± 0.01 at 15 mm and 2.89 ± 0.04 at the Bragg peak (48 mm). The influence of noise was small under the imaging conditions of this study, and we considered that the evaluation of *R*_1_ was possible with a resolution of 1 × 1 × 1 mm^3^. Here, Figure 3 illustrates the one-line δ*R*_1_ distribution profile obtained by subtracting one-line *R*_1_-values of non-irradiated NC-FG from one-line *R*_1_-values of each irradiated NC-FG. Our results confirm that the δ*R*_1_ distribution increased according to the dose, with a dose peak (Bragg peak) of approximately 48 mm. The δ*R*_1_ dose–response curves near the entrance surface (15 mm) and the Bragg peak (48 mm) were plotted in the inset of Figure 3, revealing a high linearity *R*_1_-entrance surface dose (ESD) response. Note that the horizontal axis represents the ESD. The amount of change in *R*_1_ at the Bragg peak is higher than the entrance surface. The slopes were 1.08 ± 0.02 s^−1^KGy^−1^ at the entrance surface and 3.84 ± 0.15 s^−1^KGy^−1^ at the Bragg peak, and the correlation coefficients (*R^2^*) were 0.999 and 0.994, respectively.

**Figure 4:**
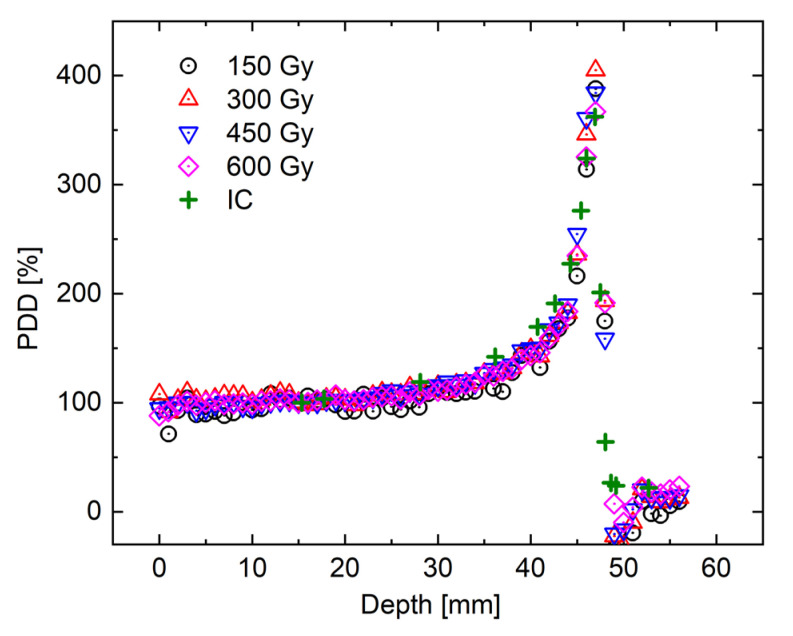
PDD curve under 290 MeV/u carbon ion beam irradiation. Comparison of the *δR*_1_ distributions with the physical dose distribution measured by the Markus IC.

Next, the percentage depth dose (PDD) curve was plotted with the ESD as 100% for each irradiated sample, and it was compared with the physical dose distribution obtained by the IC (Figure 4). Here, these values were normalized at the entrance surface. The error was omitted since it is presented in Figure 3. It was found that the physical dose distribution could be roughly reproduced at any irradiated NC-FG sample. However, before and after the Bragg peak, the *R*_1_ values were higher and lower, respectively, than the IC measurement values. This phenomenon was not shown in our previous MRI measurement conditions [34], and thus, it was considered to represent a truncation artifact due to its sharp edges [63]. Truncation artifacts occur when the MR signal obtained by finite sampling is Fourier transformed to reconstruct an image [64,65]. This artifact appears as a regular signal overshoot and undershoot in the region where the MR signal strength changes rapidly. As shown in Figure 4, the signal of *R*_1_ changed sharply at the Bragg peak. Therefore, it was considered that an overshoot occurred at the peak, and an undershoot occurred after the peak. One way to reduce truncation artifacts is to increase the matrix size of the MR image. Increasing the matrix size reduces the pixel diameter of the image. As a result, signal changes are shown more accurately and signal changes due to truncation artifacts are less noticeable. However, increasing the matrix size in the phase encoding direction even prolongs the MR scan time and lowers the SNR of the image. For example, it is considered that this experimental condition is sufficient for imaging processes, such as spread-out Bragg peak beam irradiation [66], which is closer to the actual treatment procedure as opposed to imaging the dose distribution of mono-energetic beams.

### 3.2. R_1_ vs. [Fe^3+^] Calibration Curve Using VFA and SE Methods

**Figure 5:**
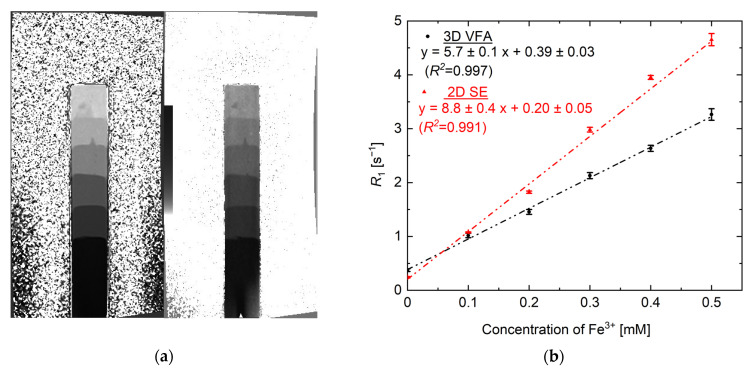
(**a**) *R*_1_ maps of the reference gel obtained from the VFA (right) and SE methods (left). (**b**) Changes in the relaxation rate *R*_1_ vs. (Fe^3+^) used as the calibration curve. Squares and triangles represent the values obtained from the VFA and SE methods, respectively. The error bars are the standard deviation (SD) in the circular region of interest (ROI) φ10 mm. The dashed line represents a linear fit to the data.

Figure 5a shows the *R*_1_ map of the reference gel (non-irradiated) with different concentrations of Fe^3+^. The results obtained by the 3D VFA data were obtained by averaging 10 slices in the slice direction in order to match with the 2D data obtained by the SE methods. It was also confirmed that the *R*_1_ change on the *R*_1_ map was due to the different Fe^3+^ concentrations in these measurements. The average *R*_1_ value obtained from the circular region of interest (ROI) φ10 mm was plotted against the concentration dependence on each Fe^3+^ concentration gel (Figure 5b). Linearity was confirmed for both the VFA and SE measurements. In contrast, the VFA results revealed a lower slope (low sensitivity characteristic) compared to the SE method. Previous studies have reported that the VFA measurements are well reproduced with other *T*_1_ measurements [54], and thus, it is considered that the difference shown in Figure 5b can be improved through the optimization of the *TR* and *FA* combination. Although further investigations on true *R*_1_ measurements are required, the high linearity *R*_1_ response is important for dose conversion when used as a dosimeter. In other words, it is shown that conversion to 3D dose distribution measurement can be performed by one conversion coefficient.

Next, we discuss the slope of Fe^+3^ dependence of *R*_1_ obtained from VFA. This value means that *R*_1_ increases the per-unit concentration (mM^−1^s^−1^). The radiation chemical yield *G*(Fe^3+^)_NC-FG_ can be calculated, as shown in the following Equation (4), by using the sensitivity characteristic at the entrance surface.

(4) $$G{\left({\mathrm{Fe}}^{3+}\right)}_{\mathrm{NC}-\mathrm{FG}}\left[{\mu \mathrm{mol}J}^{-1}]=1.08\;[s^{-1}{\mathrm{KGy}}^{-1}]÷5.7\;[s^{-1}{\mathrm{mM}}^{-1}]÷\rho \;[{\mathrm{kg}L}^{-1}\right]$$

where ρ is a density of 1.007 ± 4 $\left[{\mathrm{kg}L}^{-1}\right]$ [34]. The result is 0.188 ± 0.005 μmol/J, which is almost the same as the value of previous our reported (0.19 μmol/J) [34]. This suggests the possibility that the absolute dose could be evaluated uniquely using this radiation chemical yield even if different measurement methods and MRI equipment and the ambient environment are used.

The SNR of the 2D SE and the 3D VFA-SPGR on the *R*_1_ map in Figure 5 were 18.4 and 14.2, respectively. Furthermore, the coefficient of variation ($\mathrm{CV}\%=\left(\sigma /{\bar{R}}_{1}\right)\times 100$) of the 2D SE and the 3D VFA-SPGR were 2.0% and 3.7%, respectively. Although the 3D VFA-SPGR has a small SNR, the image quality is almost the same as the image quality obtained by the 2D SE, and the range of 256 × 256 × 40 mm can be acquired in one scan in a short time (10 min). To date, there have been no reports about whole three-dimensional dosimetry using the rapid *R*_1_(1/*T*_1_) mapping method. On the other hand, there have been some reports using the rapid measurement of *R*_2_, which is the reciprocal of the transverse relaxation time (*T*_2_), also known as the spin–spin relaxation time. For example, Cho et al. [38] attempted to acquire 3D data for *R*_2_ in a short time with a 3D fast spin echo (FSE) sequence, but signal intensity artifacts, such as blur, were prominent due to the effect of T_2_ relaxation. In addition, De Deene et al. [67] reported that several hours of scanning time were required for the acquisition of 3D data for *R*_2_ using a polymer gel dosimeter. NC-FG using 3D VFA-SPGR for *R*_1_ evaluation is effective since the measurement time is shorter compared to the polymer gel dosimetry. On the other hand, total uncertainty was not as clear as discussed for polymer gel dosimeters with low LET radiation [35]. Therefore, further experiments are required for the errors of physical chemistry characteristics, such as dose sensitivity and gel density in the case of the ion beam gel dosimetry method. An investigation into the errors of the positional setup in irradiation or imaging is also a future work.

## 4. Conclusions

In this study, we investigated the effectiveness of the rapid *T*_1_ mapping by VFA-SPGR on whole 3D dose distribution measurements for heavy ion beam irradiations. The *R*_1_ map of NC-FG after irradiation of ^12^C^6+^ 290 MeV/u obtained by rapid mapping methods was linearly increased with an increasing absorbed dose, and its *R*_1_ mapping almost represented the physical dose distribution obtained from IC. In addition, it was also found that the VFA method could accurately measure the 3D dose distribution with a 1 × 1 × 1 mm^3^ resolution that required the same scanning time (20 min) as the conventional SE method. A method for reducing truncation artifacts before and after the Bragg peak is required, and thus, our future work will focus on further optimizing the imaging conditions to mitigate these artifacts. Due to the limited time of the quality control process in clinical settings, it is necessary to reduce the working time required for the acquisition of 3D volume data of the steep dose distribution of carbon beams. While the current NC-FG dosimeter is not suitable for patient-specific dose planning in radiotherapy due to its low dose sensitivity, we think that volume data using VFA-SPGR is useful for beam performance control. The method may be applicable to other gel dosimeters that use *R*_1_ of MRI to convert the radiation dose. The development of faster quantitative *R*_1_ methods may help clinicians obtain high-quality dose maps within reasonable measurement times.

## Figures and Tables

**Table 1:** MRI conditions.

Gel Sample	Ion Beam	Reference	
Method	VFA	VFA	SE Method
*TR* (ms)	15	15	500, 3000
*TE* (ms)	3.38	3.38	2.57
*FA* (°)	7 and 36	7 and 36	
FOV (mm)	256 × 256	256 × 128	256 × 128
Matrix	256 × 256	256 × 128	256 × 128
ST (mm)	1	0.5	5
Ns	40	40	1
BW (Hz/pixel)	130	130	250
NEX	4	2	1
Scan time (s)	1219	614	444 (63 + 381)

*TR*: Repetition time, *TE*: Echo time, *FA*: Flip angle, FOV: Field of view, ST: Slice thickness, Ns: Number of slices, BW: Bandwidth, NEX: Number of excitations.

## Supplementary Materials

The following materials are available online at https://www.mdpi.com/article/10.3390/gels7040233/s1, Video S1: 3D dose distribution in NC-FG gel dosimeter obtained by VFA method.
